# A New Solution to the Matrix Equation $X-A\bar{X}B=C$

**DOI:** 10.1155/2014/543610

**Published:** 2014-07-15

**Authors:** Caiqin Song

**Affiliations:** School of Mathematical Sciences, University of Jinan, Jinan 250022, China

## Abstract

We investigate the matrix equation $X-A\bar{X}B=C$. For convenience, the matrix equation $X-A\bar{X}B=C$ is named as Kalman-Yakubovich-conjugate matrix equation. The explicit solution is constructed when the above matrix equation has unique solution. And this solution is stated as a polynomial of coefficient matrices of the matrix equation. Moreover, the explicit solution is also expressed by the symmetric operator matrix, controllability matrix, and observability matrix. The proposed approach does not require the coefficient matrices to be in arbitrary canonical form. At the end of this paper, the numerical example is shown to illustrate the effectiveness of the proposed method.

## 1. Introduction

Throughout this paper, we use *R* and *C* to denote the real number field and the complex number field. We use *A* ^*T*^, $\bar{A}$, *A* ^*H*^, and A* to denote transpose, conjugate, conjugate transpose, and the adjoint matrix of *A*, respectively. *σ*(*A*) and *λ*(*B*) are the sets of characteristic eigenvalues of matrices *A* and *B*, respectively. *I* is represented as appropriate dimension identity matrix. Moreover, for *A* ∈ *C* ^*n*×*n*^, *B* ∈ *C* ^*n*×*r*^, and *C* ∈ *C* ^*m*×*n*^, we have the following notations:

(1) $$\begin{array}{c} Q_{c}\left(A,B,n\right)=\left[\begin{array}{cccc} B & AB & \cdots & A^{n-1}B \end{array}\right],\\ Q_{o}\left(A,C,k\right)=\left[\begin{array}{c} C\\ CA\\ \vdots \\ CA^{k-1} \end{array}\right],\\ f_{A}\left(s\right)=\det\left(I-sA\right)=\alpha_{n}s^{n}+\alpha_{n-1}s^{n-1}+\cdots +\alpha_{1}s+1,\\ S_{r}\left(I,A\right)=\left[\begin{array}{ccccc} I_{r} & \alpha_{1}I_{r} & \alpha_{2}I_{r} & \cdots & \alpha_{n-1}I_{r}\\ & I_{r} & \alpha_{1}I_{r} & \cdots & \alpha_{n-2}I_{r}\\ & & & \cdots & \cdots \\ & & & I_{r} & \alpha_{1}I_{r}\\ & & & & I_{r} \end{array}\right]. \end{array}$$

In this case, *Q*_*c*_(*A*, *B*, *n*), (*A*, *B*), *Q*_*o*_(*A*, *C*, *k*), and *S*_*r*_(*I*, *A*) are named as the controllability matrix, the observability matrix, and a symmetric operator matrix, respectively.

Matrix equations are often encountered in system theory and control theory, such as Lyapunov matrix equation, Sylvester matrix equation, and so on. The traditional method is that we convert this kind of matrix equations into their equivalent forms by using the Kronecker product, however, which involves the inversion of the associated large matrix and results in increasing computation and excessive computer memory. In the field of matrix algebra, some complex matrix equations have attached much attention from many researchers since it is shown in [1]. In [2, 3], the consistence of the matrix equation $AX-\bar{X}B=C$ is related to the consimilarity of two partitioned matrices associated with the matrices *A*, *B*, and *C*. In the preceding matrix equation, $\bar{X}$ denotes the matrix obtained by taking the complex conjugate of each element of *X*. Recently, in [4] some explicit expressions of the solution to the matrix equation $AX-\bar{X}B=C$ were established by means of real representation of a complex matrix, and it is shown that there exists a unique solution if and only if $A\bar{A}$ and $B\bar{B}$ have no common eigenvalues. Yuan and Liao [5] investigated the least squares solution of the quaternion *j*-conjugate matrix equation $X-A\hat{X}B=C$ (where $\hat{X}$ denotes the *j*-conjugate of quaternion matrix *X*) with the least norm using the complex representation of a quaternion matrix, the Kronecker product of the matrices and the Moore-Penrose generalized inverse. The authors in [6] considered the matrix nearness problem associated with the quaternion matrix equation *AXA* ^*H*^ + *BYB* ^*H*^ = *C*  by means of the CCD-Q, GSVD-Q, and the projection theorem in the finite dimensional inner product space. In [7, 8], the solutions to matrix equations *X* − *AXB* = *C* and $X-A\bar{X}B=C$ have been expressed in terms of the characteristic polynomial of the matrix *A*. Song and Chen [9, 10] established the explicit solutions of the quaternion matrix equations *XF* − *AX* = *C* and $X-A\tilde{X}F=C$, where $\tilde{X}$ denote the *j*-conjugate of the quaternion matrix. Wang et al. in [11–13] investigated the extreme ranks of the solutions to the quaternion matrix equations *AXB* = *C* and *AXB* + *CYD* = *E*, respectively, the equivalence canonical form of a matrix triplet over an arbitrary division ring, solvable condition for a pair of generalized Sylvester equations, and so on. Moreover, other matrix equations such as the coupled Sylvester matrix equations and the Riccati equations have also found numerous applications in control theory. For more related introduction, see [14–19] and the references therein.

In the present paper, we investigate the polynomial solutions to the Karm-Yakubovich-conjugate matrix equation. By the Faddeev Leverrier algorithm and characteristic polynomial, we provide the explicit solutions to the well-known Karm-Yakubovich-conjugate matrix equation. In addition, the equivalent forms of the solution have been proposed.

The rest of this paper is outlined as follows. In Section 2, the polynomial solutions to the Karm-Yakubovich-conjugate matrix equation $X-A\bar{X}B=C$ are given. One of the polynomial solutions has a neat and elegant form in terms of symmetric operator matrix, controllability matrix, and observability matrix. The polynomial solution to the Karm-Yakubovich-conjugate is also proposed by the generalized Faddeev Leverrier algorithm. The example is given to show the efficiency of the proposed algorithm in Section 3.

## 2. Kalman-Yakubovich-Conjugate Matrix Equation $X-A\bar{X}B\;=\;C$

In this section, the following matrix equation is investigated:

(2) $$\begin{array}{c} X-A\bar{X}B=C, \end{array}$$

where *A* ∈ *C* ^*n*×*n*^, *B* ∈ *C* ^*p*×*p*^, and *C* ∈ *C* ^*n*×*p*^.

**Lemma 1 .**

Assume that *A* ∈ *C* ^*n*×*n*^, *B* ∈ *C* ^*p*×*p*^, and *C* ∈ *C* ^*n*×*p*^, if *X* is a solution of (2). Then for any integer *n* − 1 ≥ *k* ≥ 0, the following conclusion can be established:

(3) X(B¯B)k−(AA¯)n−kX(B¯B)n=∑j=kn−1(AA¯)j−kC(B¯B)j+∑j=kn−1(AA¯)j−k(AC¯B)(B¯B)j.

**Proof**

We prove this conclusion by mathematical induction. By postmultiplying both sides of (2) by ${\left(\bar{B}B\right)}^{n-1}$, we have

(4) $$\begin{array}{c} X{\left(\bar{B}B\right)}^{n-1}-A\bar{X}B{\left(\bar{B}B\right)}^{n-1}=C{\left(\bar{B}B\right)}^{n-1}. \end{array}$$

By taking conjugate in both sides of (2), we can get

(5) $$\begin{array}{c} \bar{X}-\bar{A}X\bar{B}=\bar{C}. \end{array}$$

By postmultiplying both sides of (5) by $B{\left(\bar{B}B\right)}^{n-1}$ and premultiplying both sides of (5) by *A*, we have

(6) $$\begin{array}{c} A\bar{X}B{\left(\bar{B}B\right)}^{n-1}-A\bar{A}X\bar{B}B{\left(\bar{B}B\right)}^{n-1}=A\bar{C}B{\left(\bar{B}B\right)}^{n-1}. \end{array}$$

Combining (4) with (6), we can obtain

(7) $$\begin{array}{c} X{\left(\bar{B}B\right)}^{n-1}-A\bar{A}X\bar{B}B{\left(\bar{B}B\right)}^{n-1}=C{\left(\bar{B}B\right)}^{n-1}+A\bar{C}B{\left(\bar{B}B\right)}^{n-1}. \end{array}$$

This implies that relation (3) holds for *k* = *n* − 1. Now we assume that relation (3) holds for *k* = *N*, (*n* − 1 ≥ *N* ≥ 1); that is,

(8) X(B¯B)N−(AA¯)n−NX(B¯B)n =∑j=Nn−1(AA¯)j−NC(B¯B)j+∑j=Nn−1(AA¯)j−N(AC¯B)(B¯B)j.

Premultiplying both sides of (8) by $A\bar{A}$, we have

(9) AA¯X(B¯B)N−(AA¯)n−N+1X(B¯B)n =∑j=Nn−1(AA¯)j−N+1C(B¯B)j+∑j=Nn−1(AA¯)j−N+1(AC¯B)(B¯B)j.

By (5), we have

(10) $$\begin{array}{c} A\bar{X}B-A\bar{A}X\bar{B}B=A\bar{C}B. \end{array}$$

Combining (10) with (2), we can obtain

(11) $$\begin{array}{c} X-A\bar{A}X\bar{B}B=C+A\bar{C}B. \end{array}$$

According to (11), it is easy to obtain

(12) $$\begin{array}{c} X{\left(\bar{B}B\right)}^{N-1}-A\bar{A}X{\left(\bar{B}B\right)}^{N}=\left(C+A\bar{C}B\right){\left(\bar{B}B\right)}^{N-1}. \end{array}$$

Combining (9) with (12), we can obtain

(13) X(B¯B)N−1−(AA¯)n−N+1X(B¯B)n =∑j=N−1n−1(AA¯)j−(N−1)C(B¯B)j  +∑j=N−1n−1(AA¯)j−(N−1)(AC¯B)(B¯B)j.

This implies that relation (3) holds for *k* = *N* − 1. So the conclusion is true.

Define

(14) ∏k(A,C,B)=∑j=kn−1(AA¯)j−kC(B¯B)j+∑j=kn−1(AA¯)j−k(AC¯B)(B¯B)j,

and then equality (3) can be compactly written as

(15) $$\begin{array}{c} X{\left(\bar{B}B\right)}^{k}-{\left(A\bar{A}\right)}^{n-k}X{\left(\bar{B}B\right)}^{n}=\underset{k}{\prod }\left(A,C,B\right). \end{array}$$

Let

(16) $$\begin{array}{c} f_{A\bar{A}}\left(s\right)=\det\left(sI-A\bar{A}\right)=\alpha_{n}+\alpha_{n-1}s+\cdots +\alpha_{0}s^{n}, \end{array}$$

and then

(17) $$\begin{array}{c} {\tilde{f}}_{\left(A\bar{A}\right)}\left(s\right)=\det\left(I-sA\bar{A}\right)=\alpha_{0}+\alpha_{1}s+\cdots +\alpha_{n}s^{n}. \end{array}$$

It follows from (15), (16), and (17) that we have

(18) ∑k=0n−1αk[X(B¯B)k−(AA¯)n−kX(B¯B)n] =∑k=0n−1[αkX(B¯B)k−αk(AA¯)n−kX(B¯B)n]+αn[X(B¯B)n−X(B¯B)n] =∑k=0nαkX(B¯B)k−∑k=0nαk(AA¯)n−kX(B¯B)n =Xf~(AA¯)(B¯B)−fAA¯(AA¯)X(B¯B)n =Xf~(AA¯)(B¯B).

On the other hand,

(19) $$\begin{array}{c} \overset{n-1}{\underset{k=0}{\sum }}\alpha_{k}\left[X{\left(\bar{B}B\right)}^{k}-{\left(A\bar{A}\right)}^{n-k}X{\left(\bar{B}B\right)}^{n}\right]=\overset{n-1}{\underset{k=0}{\sum }}\alpha_{k}\underset{k}{\prod }\left(A,C,B\right). \end{array}$$

Now, we define

(20) $$\begin{array}{c} \prod \left(A,C,B\right)=\overset{n-1}{\underset{k=0}{\sum }}\alpha_{k}\underset{k}{\prod }\left(A,C,B\right). \end{array}$$

It is obvious that ∏(*A*, *C*, *B*) is a polynomial of the matrices *A*, *B*, and *C*. This polynomial is defined by the coefficient matrices and the characteristic polynomial of $A\bar{A}$. That is to say, for each Kalman-Yakubovich-conjugate equation of the form (2), there is a uniquely determined polynomial ∏(*A*, *C*, *B*) of its coefficients matrices. Therefore, we obtain the following equation:

(21) $$\begin{array}{c} X{\tilde{f}}_{A\bar{A}}\left(\bar{B}B\right)=\prod \left(A,C,B\right). \end{array}$$

**Theorem 2 .**

If *ηγ* ≠ 1, for any $\eta \in \lambda \left(A\bar{A}\right)$ and $\gamma \in \lambda \left(\bar{B}B\right)$, then (21) is equivalent to (2).

**Proof**

From the abovementioned argument, it is shown that (2) implies (21). Now we prove that (21) implies (2) when *ηγ* ≠ 1 for any $\eta \in \lambda (A\bar{A})$ and $\gamma \in \lambda (\bar{B}B)$. Now suppose that *X* is a solution of (21), we can obtain

(22) Xf~AA¯(B¯B)−AA¯XB¯Bf~AA¯(B¯B)  =(X−AA¯XB¯B)f~AA¯(B¯B)  =∏(A,C,B)−AA¯∏(A,C,B)B¯B.

In addition, we have

(23) ∏(A,C,B)−AA¯∏(A,C,B)B¯B =∑k=0n−1αk[∑j=kn−1(AA¯)j−kC(B¯B)j+∑j=kn−1(AA¯)j−k(AC¯B)(B¯B)j]  −∑k=0n−1αk[∑j=kn−1(AA¯)j−k+1C(B¯B)j+1+∑j=kn−1(AA¯)j−k+1(AC¯B)(B¯B)j+1] =∑k=0n−1αk  ×{∑j=kn−1[(AA¯)j−kC(B¯B)j−(AA¯)j−k+1C(B¯B)j+1]+[(AA¯)j−k(AC¯B)(B¯B)j−(AA¯)j−k+1(AC¯B)(B¯B)j+1]} =∑k=0n−1αk{[C(B¯B)k−(AA¯)n−kC(B¯B)n]+[(AC¯B)(B¯B)k−(AA¯)n−k(AC¯B)(B¯B)n]} =Cf~AA¯(B¯B)−fAA¯(AA¯)C(B¯B)n  +(AC¯B)f~AA¯(B¯B)−fAA¯(AA¯)(AC¯B)(B¯B)n =Cf~AA¯(B¯B)+(AC¯B)f~AA¯(B¯B).

Combining this with (22) gives

(24) $$\begin{array}{c} \left(X-A\bar{A}X\bar{B}B\right){\tilde{f}}_{A\bar{A}}\left(\bar{B}B\right)=\left(C+A\bar{C}B\right){\tilde{f}}_{A\bar{A}}\left(\bar{B}B\right). \end{array}$$

Since *ηγ* ≠ 1 for any $\eta \in \lambda (A\bar{A})$ and $\gamma \in \lambda (\bar{B}B)$, the matrix ${\tilde{f}}_{A\bar{A}}(\bar{B}B)$ is nonsingular. Hence, it is obtained from (24) that (24) implies (2). With the above two aspects, the conclusion is proved.

The following theorem gives a result on the unique solution of the Kalman-Yakubovich-conjugate matrix equation.

**Theorem 3 .**

If *ηγ* ≠ 1 for any $\eta \in \lambda (A\bar{A})$ and $\gamma \in \lambda (\bar{B}B)$, then the unique solution of the matrix equation (2) is

(25) $$\begin{array}{c} X=\prod \left(A,C,B\right){\left[{\tilde{f}}_{A\bar{A}}\left(\bar{B}B\right)\right]}^{-1}, \end{array}$$

which is a polynomial of matrices *A*, *B*, and *C*.

**Proof**

Firstly, we assume that the characteristic polynomial of ${\tilde{f}}_{A\bar{A}}(\bar{B}B)$ be $f_{{\tilde{f}}_{A\bar{A}}(\bar{B}B)}(s)=\sum_{i=0}^{p}\beta_{i}s^{i}$. Because ${\tilde{f}}_{A\bar{A}}(\bar{B}B)$ is nonsingular, it is shown that *β*_0_ ≠ 0. It follows from Cayley-Hamilton's theorem that

(26) $$\begin{array}{c} f_{{\tilde{f}}_{A\bar{A}}(\bar{B}B)}\left({\tilde{f}}_{A\bar{A}}\left(\bar{B}B\right)\right)=\overset{p}{\underset{i=1}{\sum }}\beta_{i}{\left[{\tilde{f}}_{A\bar{A}}\left(\bar{B}B\right)\right]}^{i}+\beta_{0}I=0. \end{array}$$

This relation implies that

(27) $$\begin{array}{c} {\tilde{f}}_{A\bar{A}}\left(\bar{B}B\right)\left\{\overset{p}{\underset{i=1}{\sum }}\beta_{i}{\left[{\tilde{f}}_{A\bar{A}}\left(\bar{B}B\right)\right]}^{i-1}\right\}=-\beta_{0}I. \end{array}$$

Therefore, we have

(28) $$\begin{array}{c} {\left[{\tilde{f}}_{A\bar{A}}\left(\bar{B}B\right)\right]}^{-1}=-\frac{1}{\beta_{0}}\overset{p}{\underset{i=1}{\sum }}\beta_{i}{\left[{\tilde{f}}_{A\bar{A}}\left(\bar{B}B\right)\right]}^{i-1}, \end{array}$$

which is a polynomial of ${\tilde{f}}_{A\bar{A}}(\bar{B}B)$. Since ${\tilde{f}}_{A\bar{A}}(\bar{B}B)$ is a polynomial of $\bar{B}B$, it is easily known that ${\left[{\tilde{f}}_{A\bar{A}}(\bar{B}B)\right]}^{-1}$ is a polynomial of  $\bar{B}B$. So we can see that $\prod (A,C,B){\left[{\tilde{f}}_{A\bar{A}}(\bar{B}B)\right]}^{-1}$ is a polynomial of matrices *A*, *B*, and *C*.

Next, we give two equivalent forms of the solution to the matrix equation (2). In order to obtain the unique solution of the matrix equation (2), only the coefficients of characteristic polynomial of $A\bar{A}$ are required. Firstly, the so-called generalized Faddeev-Leverrier algorithm [20] is stated as the following relations:

(29) Uk=Uk−1(AA¯)+αkI, U0=I,  k=1,2,…,n.αk=trace(Uk−1AA¯)k, α0=1,  k=1,2,…,n,

where *α*_*i*_, *i* = 0,1, 2,…, *n* − 1, are the coefficients of the characteristic polynomial of the matrix $A\bar{A}$, and *U*_*i*_, *i* = 0,1,…, *n* − 1, are the coefficient matrices of the adjoint matrix ${\left(sI_{n}-A\bar{A}\right)}^{*}$.

So we have the following theorems.

**Theorem 4 .**

Given the matrices *A* ∈ *C* ^*n*×*n*^, *B* ∈ *C* ^*p*×*p*^, and *C* ∈ *C* ^*n*×*p*^, let

(30) $$\begin{array}{c} {\tilde{f}}_{A\bar{A}}\left(s\right)=\det\left(I-sA\bar{A}\right)=\alpha_{n}s^{n}+\cdots +\alpha_{1}s+\alpha_{0},\;\alpha_{0}=1,\\ {\left(I-sA\bar{A}\right)}^{*}=U_{n-1}s^{n-1}+\cdots +U_{1}s+U_{0}. \end{array}$$

(1) If *X* is a solution to (2), then
  (31) $$\begin{array}{c} X{\tilde{f}}_{A\bar{A}}\left(\bar{B}B\right)=\overset{n-1}{\underset{j=0}{\sum }}U_{j}C{\left(\bar{B}B\right)}^{j}+\overset{n-1}{\underset{j=0}{\sum }}U_{j}\left(A\bar{C}B\right){\left(\bar{B}B\right)}^{j}. \end{array}$$
(2) If the matrix ${\tilde{f}}_{A\bar{A}}(\bar{B}B)$ is nonsingularity, then the matrix equation (2) has the unique solution; that is,
  (32) $$\begin{array}{c} X=\left[\overset{n-1}{\underset{j=0}{\sum }}U_{j}C{\left(\bar{B}B\right)}^{j}+\overset{n-1}{\underset{j=0}{\sum }}U_{j}\left(A\bar{C}B\right){\left(\bar{B}B\right)}^{j}\right]{\left[{\tilde{f}}_{A\bar{A}}\left(\bar{B}B\right)\right]}^{-1}. \end{array}$$

**Proof**

By applying (29), we can obtain the following expression:

(33) U0=I,U1=α1I+AA¯,U2=α2I+α1AA¯+(AA¯)2,⋮Un−1=αn−1I+αn−2AA¯+⋯+(AA¯)n−1.

Meanwhile, the above formula can be stated as

(34) $$\begin{array}{c} U_{j}=\overset{j}{\underset{k=0}{\sum }}\alpha_{k}{\left(A\bar{A}\right)}^{j-k},\;\alpha_{0}=1,\;\;j=1,2,\ldots ,n-1. \end{array}$$

Thus, it is easy to know

(35) ∑k=0n−1 ‍∑j=kn−1αk(AA¯)j−kC(B¯B)j  +∑k=0n−1 ‍∑j=kn−1αk(AA¯)j−k(AC¯B)(B¯B)j =∑j=0n−1[∑k=0jαk(AA¯)j−k]C(B¯B)j  +∑j=0n−1[∑k=0jαk(AA¯)j−k](AC¯B)(B¯B)j =∑j=0n−1UjC(B¯B)j+∑j=0n−1Uj(AC¯B)(B¯B)j.

Thus, we can easily obtain the conclusions.

**Theorem 5 .**

Suppose the matrices *A* ∈ *C* ^*n*×*n*^, *B* ∈ *C* ^*p*×*p*^, and *C* ∈ *C* ^*n*×*p*^.

(1) Let *X* be a solution of (2), thus,
  (36) Xf~AA¯(B¯B)=Qc(AA¯,C,n)Sp(I,AA¯)Qo(B¯B,I,n)+Qc(AA¯,A,n)Sp(I,AA¯)Qo(B¯B,C¯B,n).
(2) Let *λμ* ≠ 1 for any *λ*, $\mu \in \sigma (A\bar{A})$; thus, the matrix equation (2) has a unique solution
  (37) X=[Qc(AA¯,C,n)Sp(I,AA¯)Qo(B¯B,I,n)+Qc(AA¯,A,n)Sp(I,AA¯)Qo(B¯B,C¯B,n)]×[f~AA¯(B¯B)]−1.

**Proof**

In view of relation (33), it is obvious that

(38) $$\begin{array}{c} \left[\begin{array}{cccc} U_{0}C & U_{1}C & \cdots & U_{n-1}C \end{array}\right]=Q_{c}\left(A\bar{A},C,n\right)S_{p}\left(I,A\bar{A}\right).\\ \left[\begin{array}{cccc} U_{0}A & U_{1}A & \cdots & U_{n-1}A \end{array}\right]=Q_{c}\left(A\bar{A},A,n\right)S_{p}\left(I,A\bar{A}\right). \end{array}$$

Then it is easy to obtain that

(39) ∑j=0n−1UjC(B¯B)j  =[U0CU1C⋯Un−1C]Qo(B¯B,I,n)  =Qc(AA¯,C,n)Sp(I,AA¯)Qo(B¯B,I,n),∑j=0n−1Uj(AC¯B)(B¯B)j   =[U0AU1A⋯Un−1A]Qo(B¯B,C¯B,n)   =Qc(AA¯,A,n)Sp(I,AA¯)Qo(B¯B,C¯B,n).

Combining this with Theorem 4, we complete the proof.

On the basis of the above results, we have the following corollary on the solution of the Stein-conjugate matrix equation $X-A\bar{X}A^{T}=C$.

**Corollary 6 .**

Let the matrices *A* ∈ *C* ^*n*×*n*^ and *C* ∈ *C* ^*n*×*n*^ be given. If the matrix $A\bar{A}$ is Schur stable, then the unique solution of the matrix equation $X-A\bar{X}A^{T}=C$ is expressed as

(40) $$\begin{array}{c} X=\prod \left(A,C,A^{T}\right){\left[{\tilde{f}}_{A\bar{A}}\left(A^{H}A^{T}\right)\right]}^{-1}, \end{array}$$

which is a polynomial of matrices *A* and *C*.

## 3. Numerical Example

**Example 1 .**

Here we give an example for computing the solution to matrix equation $X-A\bar{X}B=C$.

The parametric matrix can be written as follows:

(41) A=[i0241+i123i0],  B=[1i−i2],C=[−1−i−1+4i−8−13i−19+11i−11−i−1+14i].

It is easy to check that *ηγ* ≠ 1, for any $\eta \in \lambda (A\bar{A})$ and $\gamma \in \lambda (\bar{B}B)$. So we can see that the above matrix equation has a unique solution.

The following result can be obtained by some simple computation:

(42) f(AA¯)(s)=s3−11s2+43s−401,f~(AA¯)(s)=−401s3+43s2−11s+1.

Thus *α*_0_ = 1, *α*_1_ = −11, and *α*_2_ = 43; in addition, we have

(43) $$\begin{array}{c} f_{\left(A\bar{A}\right)}\left(\bar{B}B\right)=\left[\begin{array}{cc} 1161 & 3090i\\ 3090i & -8109 \end{array}\right],\\ {\left[f_{\left(A\bar{A}\right)}\left(\bar{B}B\right)\right]}^{-1}=\left[\begin{array}{cc} -0.0607 & -0.0231i\\ -0.0231i & 0.0087 \end{array}\right]. \end{array}$$

So we have the following matrix expression:

(44) ∏0(A,C,B)=C+AC¯B+(AA¯)(C+AC¯B)(B¯B)+(AA¯)2(C+AC¯B)(B¯B)2,∏1(A,C,B)=(C+AC¯B)(B¯B)+(AA¯)(C+AC¯B)(B¯B)2,∏2(A,C,B)=(C+AC¯B)(B¯B)2,α0∏0(A,C,B)+α1∏1(A,C,B)+α2∏2(A,C,B)  =[1161+6180i−16218+3090i3090+5412i−14289+8109i3090i−8109].

Therefore, it follows from Theorem 3 that the unique solution of (2) is

(45) X=[α0∏0(A,C,B)+α1∏1(A,C,B)+α2∏2(A,C,B)] ×[f(AA¯)(B¯B)]−1=[1.0000+0.0000i2.0000+0.0000i−0.0000+2.0000i1.0000−1.0000i0.0000i1.0000].

## 4. Conclusion

The well-known Karm-Yakubovich matrix equation and the generalized discrete Sylvester matrix equation have many important applications in control theory and system theory. As the generalization of the above matrix equations, in this paper we have proposed polynomial solutions to the Karm-Yakubovich-conjugate matrix equation. Different from the other approaches, the approach in the current paper does not require transformation of the coefficient matrices into any canonical form. The solutions are stated as a polynomial of parameters of the matrix equation. All the coefficient matrices are not restricted to be in any canonical form. Meanwhile, an equivalent form of the solutions to the Karm-Yakubovich-conjugate matrix equation has been expressed in terms of controllability matrix associated with *A*, *B*, and *C* and observability matrix associated with *A* and *B*. Such a feature may bring greater convenience and advantages to some problems related to the Karm-Yakubovich-conjugate matrix equation. At the end of the paper, the numerical experiment is done to illustrate the performance of the proposed method. From the discussion in our paper, on one hand, one can observe that the solutions to the Karm-Yakubovich-conjugate matrix equation are crucial as the theoretical basis of the development of many kinds of other matrix equations and deserve further investigation in the future and, on the other hand, as the theoretical generalization of the well-known Karm-Yakubovich matrix equation, it may be helpful for future control applications.
